# Knockdown of SF-1 and RNF31 Affects Components of Steroidogenesis, TGFβ, and Wnt/β-catenin Signaling in Adrenocortical Carcinoma Cells

**DOI:** 10.1371/journal.pone.0032080

**Published:** 2012-03-09

**Authors:** Anna Ehrlund, Philip Jonsson, Lise-Lotte Vedin, Cecilia Williams, Jan-Åke Gustafsson, Eckardt Treuter

**Affiliations:** 1 Center for Biosciences, Department of Biosciences and Nutrition, Karolinska Institutet, Huddinge, Sweden; 2 Center for Nuclear Receptors and Cell Signaling, Department of Biology and Biochemistry, University of Houston, Houston, Texas United States of America; 3 Department of Medicine, Karolinska Institutet, Huddinge, Sweden; National Cancer Center, Japan

## Abstract

The orphan nuclear receptor Steroidogenic Factor-1 (SF-1, NR5A1) is a critical regulator of development and homeostasis of the adrenal cortex and gonads. We recently showed that a complex containing E3 ubiquitin ligase RNF31 and the known SF-1 corepressor DAX-1 (NR0B1) interacts with SF-1 on target promoters and represses transcription of steroidogenic acute regulatory protein (StAR) and aromatase (CYP19) genes. To further evaluate the role of SF-1 in the adrenal cortex and the involvement of RNF31 in SF-1-dependent pathways, we performed genome-wide gene-expression analysis of adrenocortical NCI-H295R cells where SF-1 or RNF31 had been knocked down using RNA interference. We find RNF31 to be deeply connected to cholesterol metabolism and steroid hormone synthesis, strengthening its role as an SF-1 coregulator. We also find intriguing evidence of negative crosstalk between SF-1 and both transforming growth factor (TGF) β and Wnt/β-catenin signaling. This crosstalk could be of importance for adrenogonadal development, maintenance of adrenocortical progenitor cells and the development of adrenocortical carcinoma. Finally, the SF-1 gene profile can be used to distinguish malignant from benign adrenocortical tumors, a finding that implicates SF-1 in the development of malignant adrenocortical carcinoma.

## Introduction

The adrenal cortex is the main site for synthesis of mineralocorticoids, glucocorticoids and adrenal androgens and is thus of critical importance for a wide variety of physiological processes including salt balance, immune system and stress responses. The fetal adrenal cortex has a hormone-secretion profile distinct from the adult cortex and it is not until after birth that the adult cortex, with its three distinct functional and morphological zones, forms. The fetal zone then regresses by apoptosis. It is believed that the subcapsular cell layer of the cortex contains adrenocortical progenitor cells responsible for the regenerative capacity of the cortex. The progenitors characteristically express the transcription factors Steroidogenic Factor-1 (SF-1) and DAX-1 (NR0B2), both belonging to the nuclear receptor family (see [1] for a recent review).

Adrenocortical carcinoma (ACC) is a rare disease with an incidence of approximately one per million per year. It has a poor prognosis and no efficient therapies exist. ACC is believed to develop in a multistep process where normal cells first transform into benign tumors. Rearrangements in the benign tumor sometimes take place and turn it into a malignant, invasive cancer [2]. Childhood adrenocortical tumors (ACT) are rare, representing between 0.05–0.2% of all pediatric cancers. The children usually present symptoms before five years of age. Childhood ACTs are believed to represent a failure of the fetal adrenal tissue to regress fully. The tumors often overexpress IGF2 and also carry other characteristics of the fetal adrenal cortex [3].

An interesting feature of childhood ACTs is their overexpression of SF-1 [4], [5]. SF-1 is a nuclear receptor almost exclusively expressed in the steroidogenic tissues of the hypothalamic-pituitary-adrenal/gonadal axis [6], [7]. SF-1 is also crucial during the embryonic development of the adrenal gland [8] and gonads [9], a point highlighted by the fact that SF-1 knockout mice lack both adrenals and gonads [10], [11]. Functionally, SF-1 is known to transcriptionally regulate the expression of genes involved in steroid hormone synthesis and cellular cholesterol homeostasis [12]. However, less is known about SF-1's mechanisms of action and target genes in proliferation and differentiation during development and cancer [13].

Mechanistically, SF-1 binds as a monomer to specific response elements in the promoters of its target genes. Bound SF-1 recruits either corepressor complexes, which put the gene in a silent state, or coactivator complexes, which activate transcription by altering histone modifications and recruiting the general transcription machinery including RNA polymerase II [14], [15], [16]. Structural studies have shown that SF-1 has a ligand-binding pocket that can accommodate phospholipids [17], [18], [19] and the search for a natural ligand is ongoing. Sphingosine has been shown to act as a natural antagonist ligand to SF-1 [20] and adrenocorticotropic hormone (ACTH), which raises intracellular cAMP levels and induces steroidogenesis, was shown to increase sphingosine catabolism. As the sphingosine concentration drops, the authors speculate that a natural agonist ligand binds SF-1 instead and activates transcription of target genes [21]. This would be an additional mechanism for ACTH to induce steroidogenesis, complementary to activation of the cAMP-binding transcription factors (CREB/CREM) that also regulate expression of steroidogenic enzymes.

Post-translational modifications of SF-1 are known to play an important role in regulating its transcriptional actions. Phosphorylation of residues in the hinge region by kinases in the MAPK pathway enhances SF-1-dependent transcription [22] while SUMOylation of lysine residues in the same region can repress SF-1 [23], [24], [25], [26]. What signals induce SUMOylation of SF-1 remains to be elucidated. We recently described how repression of SF-1 via the orphan receptor/corepressor DAX-1 is reliant on the putative E3 ubiquitin ligase RNF31 (ZIBRA, PAUL, HOIL). RNF31 can ubiquitinate DAX-1 and it seems that the ubiquitination is important for the assembly of a corepressor complex containing DAX-1, SMRT and RNF31 on StAR and aromatase (CYP19) promoters. The complex does not co-occupy the promoter together with active RNA pol II, indicating that transcription is tuned down [14].

In an effort to elucidate the roles of SF-1 and RNF31 in adrenal steroidogenesis and adrenocortical carcinoma on a genome-wide level, we performed siRNA-mediated knockdown of SF-1 or RNF31 ± cAMP in the adrenocortical carcinoma cell line NCI-H295R. Analysis of the differentially expressed genes indicates a systemic role for SF-1 in the adrenal cortex, affecting differentiation, proliferation and steroidogenesis and establishes RNF31 as an important regulator of adrenal steroidogenesis.

## Results and Discussion

### SF-1 affects a multitude of genes in adrenocortical carcinoma cells

The human adrenocortical carcinoma cell line NCI-H295R produces the major adrenal steroid hormones, including glucocorticoids and dehydroepiandrosterone (DHEA), and has functional SF-1/DAX-1 and CREB/CREM-pathways. To our knowledge this is the only human adrenocortical carcinoma cell line available with all these properties. In this cell line we used an RNAi-silencing approach combined with raised intracellular cAMP levels following forskolin treatment to assess the effects of the two main pathways of steroidogenic gene induction.

**Figure 1:**
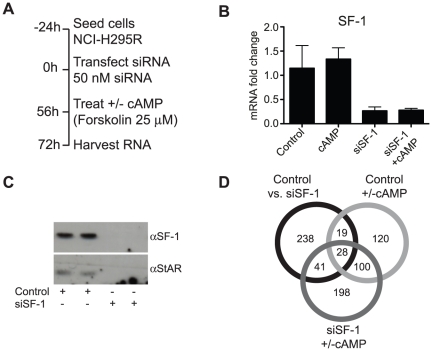
Analysis of SF-1 RNAi +/− cAMP-treated H295R cells. (**A**) Experimental design. (**B**) qPCR of Control and siSF ± cAMP-treated cells show approximately 70% efficiency of SF-1 RNAi-mediated knockdown on mRNA level. (**C**) Western blot showing almost complete knockdown of SF-1 protein after RNAi-treatment. StAR, a protein under transcriptional control of SF-1, is also depleted, showing efficiency of knockdown on target gene level. (**D**) Venn diagram showing overlap between the different groups of differentially expressed genes.

Cells were seeded 24 h before transfection with SF-1 siRNA oligos. Optimizing experiments had shown that 72 h of siRNA treatment had a good silencing effect. We added forskolin to indicated samples 16 h before harvest (Figure 1A). The RNAi treatment knocked down SF-1 mRNA about 70% (Figure 1B) and protein levels even further (Figure 1C). We also saw a dramatic decrease of the known SF-1 target gene StAR's protein levels (Figure 1C), indicating that the achieved knockdown was sufficient to detect changes at target-gene level.

After quality control, samples were analyzed on microarrays to reveal genome-wide effects of SF-1 knockdown (Control vs. siSF-1), effect of cAMP treatment (Control +/−cAMP) and effect of cAMP treatment when SF-1 was knocked down (siSF-1 +/− cAMP). After data processing, a total of 326, 367 and 267 genes were defined as differentially regulated in the Control vs. siSF-1, the siSF-1 +/− cAMP and the Control +/− cAMP comparisons, respectively. Lists of the 35 most upregulated and 35 most downregulated genes for each comparison can be found in the supplementary materials (Tables S1, 2, 3, 4, 5, 6). Notably, SF-1 is the second most downregulated gene in the siSF-1 microarray.

The overlap (Figure 1D) between the cAMP-treated and SF-1-knockdown samples was 48 genes (Table 1), representing 15% of the total number of genes found regulated by SF-1 knockdown. Both SF-1 and cAMP are considered inducers of steroidogenesis, which should result in upregulation of steroidogenic genes in the cAMP-treated and downregulation in the siSF-1. This was true for 11 out of 48 genes, not all of them clearly connected to steroidogenesis. The overlap between the siSF-1 +/− cAMP comparison and the Control +/− cAMP was 128 genes, which corresponds to 48% of the Control + cAMP-regulated genes – a large and expected overlap. Between the siSF-1 and siSF-1 + cAMP sample, there was an overlap of 69 genes representing 22% of the siSF-1 genes. The overlap between all three experiments was 28 genes.

**Table 1:** Genes changed by both siSF-1 and cAMP treatment.

siSF-1 downregulated			siSF-1 upregulated		
Gene	siSF-1	cAMP	Gene	siSF-1	cAMP
STAR	−1.47	2.30	TFPI2	0.74	3.03
RHOB	−1.09	1.46	GNG11	0.79	1.63
CSN1S1	−0.99	2.13	SLC12A2	0.79	1.42
SCARB1	−0.91	1.43	DNER	0.57	1.26
CYP11A1	−0.85	1.05	ENPP2	0.98	1.08
ITGA9	−0.85	1.02	PBX1	0.67	0.91
DUSP16	−0.84	0.96	ATP1B3	1.29	0.78
CYP17A1	−0.81	1.90	OSBPL6	0.82	−0.72
HSPD1	−0.77	1.10	UACA	0.57	−0.75
MOBKL3	−0.76	1.47	ZNRF3	0.87	−0.75
C2CD2	−0.68	1.00	PIP4K2A	1.28	−0.78
TMEM200A	−1.34	−1.03	NKD1	1.57	−0.79
SULT2A1	−0.83	−1.50	AXIN2	1.07	−0.85
SV2B	−0.71	−1.34	SLC7A8	1.37	−0.87
SMS	−0.61	−1.07	KCTD12	0.70	−0.98
SLC35D1	−0.63	−1.04	ANGPTL2	0.85	−1.06
PDE2A	−0.84	−0.90	CCND2	1.36	−1.16
KLHL5	−0.77	−1.01	ACPL2	0.59	−1.36
HSPB7	−0.70	−0.84	APCDD1	1.24	−1.42
GSTA2	−0.79	−1.31	FGF13	1.32	−1.48
GSTA1	−0.80	−1.36	PRSS23	0.80	−1.49
FIBCD1	−0.67	−1.10	ANKFN1	1.83	−2.25
CCDC141	−0.73	−1.23			
ALDH1A1	−0.57	−1.08			
AGTR1	−0.67	−0.77			

Values given in log_2_ (fold change).

Knockdown of SF-1 upregulates approximately the same number of genes as it downregulates and though an unknown number of these genes are indirect targets it seems plausible that SF-1, as many other NRs including LRH-1, has a repressive effect on some of its target genes.

### SF-1 and cAMP regulate steroidogenic target genes

**Figure 2:**
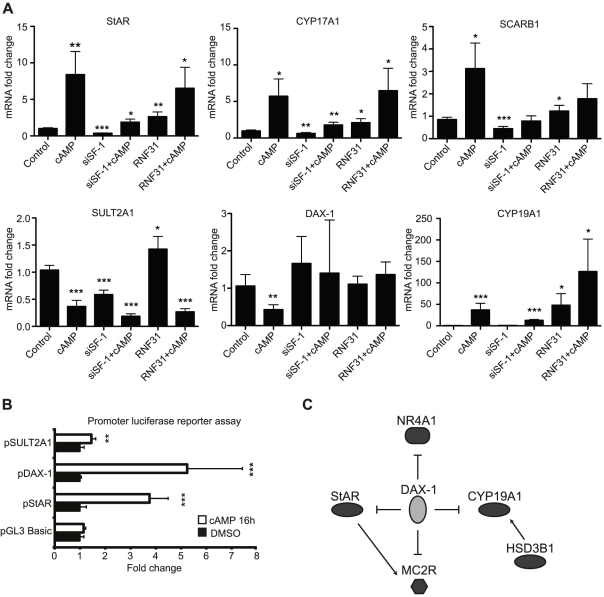
Confirmation of steroidogenic target genes differentially expressed in microarrays and promoter assays of StAR, DAX-1 and SULT2A1 proximal promoters after cAMP treatment and flow-cytometry assay of siSF-1 treated cells. (**A**) qPCR showing mRNA levels of six steroidogenic target genes. (**B**) Luciferase reporter assays of StAR, DAX-1 and SULT2A1 promoters after 16 h of ± cAMP treatment show that the down regulation of DAX-1 and SULT2A1 mRNA levels after cAMP-treatment cannot be recreated with the proximal 1 kb of the promoters alone. (**C**) Expression targets of DAX-1 differentially expressed in the Control vs. siRNF31 microarray as identified by Pathway Studio. Error bars show standard deviation. Statistical analysis done with Student's t-test (*p<0.05; **p<0.01; ***p<0.001).

To further confirm the validity of the knockdown methodology and the microarray cut-off limits, we ran qPCR on known SF-1 target genes. In a recent review, Hoivik *et al.* [27] lists adrenal SF-1 target genes. We compared this list to our microarray results and found 6 of the 13 mentioned genes regulated by SF-1 and 5 by cAMP treatment, and performed verifications using qPCR (Figure 2A). A 100% overlap is not to be expected since the target genes in the literature are reported from different systems (cell lines or tissues) and species. Overall, the genes known to be induced by SF-1 correlate quite well with those downregulated by SF-1 knockdown in our experiments.

In our system we did not see effects of SF-1 knockdown on DAX-1 basal expression, neither in the microarray analysis nor qPCR experiments, even though the standard deviation in the qPCR data is quite large (the tendency, if any, is rather upregulation in response to SF-1 knockdown). This is surprising, as previous studies using promoter reporter assays and overexpression have identified SF-1 response elements in the DAX-1 promoter [28], [29]. Those appear active in the developing mouse gonad [30] and DAX-1 levels are decreased in SF-1 knockout mice [31]. However, it is plausible that SF-1 could regulate DAX-1 expression in a tissue-specific manner. In that case, it may well be that DAX-1 is independent of SF-1 in the state represented by the NCI-H295R cells or that compensatory mechanisms step in when SF-1 expression is diminished.

The steroidogenic genes tested are also targets of cAMP signaling and thus serve the same confirmatory purpose for the Control +/− cAMP array. The genes are generally upregulated with two exceptions, DAX-1 and SULT2A1, which are distinctly downregulated by forskolin treatment. We tested the effect of raised cAMP levels on proximal (1 kb) promoter reporter constructs of StAR, DAX-1 and SULT2A1 in the NCI-H295R cells. Interestingly, while the endogenous gene is clearly downregulated, the proximal promoters were induced by cAMP (Figure 2B). This indicates that the effect is mediated either by elements upstream of the cloned promoter fragments or that chromatin remodeling events not captured by the artificial promoter are involved. Manna *et al.* reported on the inhibitory effect of cAMP on DAX-1 mRNA and protein expression and showed that it could be abolished by inhibiting either PKA or PKC and that it required *de novo* protein synthesis [32], and therefore should not be a direct effect of cAMP activating a transcription factor. To our knowledge, the inhibitory effect of cAMP on SULT2A1 has not been reported before. It seems to be a tissue-specific effect as raised cAMP levels in hepatocytes cause an increase in SULT2A1 expression that is dependent on the nuclear receptor CAR [33]. Whether or not the cAMP-dependent repression of SULT2A1 and DAX-1 follows similar mechanisms and what those mechanisms may be remains to be investigated.

### RNF31 is a regulator of adrenal steroidogenic processes

**Figure 3:**
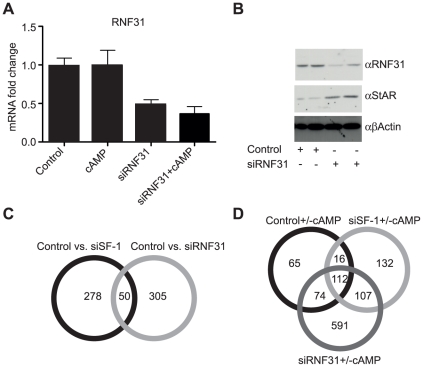
Analysis of differentially expressed genes in siRNF31 ± cAMP-treated H295R cells. (**A**) qPCR showing approximately 60% efficiency of RNF31 RNAi-treatment on mRNA level. (**B**) Western blot showing efficient knockdown of RNF31 protein and upregulation of RNF31 target StAR in RNF31 RNAi-treated H295R cells. (**C**) Venn diagram showing overlap of differentially expressed genes in siSF-1 and siRNF31 microarrays. (**D**) Venn diagram overlap among the differentially expressed genes in all cAMP-treated samples.

We recently identified RNF31 as a member of a corepressor complex including SMRT and DAX-1 that is involved in repression of SF-1-mediated transcription [14]. To investigate the genome-wide effects of RNF31 we used siRNA to knock down its expression in the NCI-H295R cell line. The knockdown of RNF31 was less efficient than that of SF-1, about 60% at the mRNA level (Figure 3A) and with some residual protein remaining (Figure 3B). However, the protein level of the target gene StAR was notably increased upon knockdown of RNF31, indicating that the achieved level was sufficient to detect putative target genes (Figure 3B). Using the same experimental outline as for the SF-1 experiment, we observed that RNF31 knockdown resulted in 355 regulated genes. The overlap with the SF-1 knockdown is 50 genes (Figure 3C and Table 2) and of those, 9 genes were upregulated in the siRNF31 while downregulated in the siSF-1 sample (Table 2).

**Table 2:** Genes changed by both siSF-1 and siRNF31 treatment.

	siSF-1	siRNF31
**siSF-1 downregulated**		
STAR	−1.47	0.89
CSN1S1	−0.99	0.71
SCARB1	−0.91	0.42
ITGA9	−0.85	0.76
DUSP16	−0.84	0.48
SULT2A1	−0.83	0.45
CYP17A1	−0.81	1.12
MC2R	−0.61	0.88
OR4S2	−0.58	0.47
NCRNA00086	−0.99	−0.45
ZBTB7C	−0.85	−0.55
KLHL5	−0.77	−0.55
FNDC3B	−0.64	−0.73
SLC35D1	−0.63	−0.52
FAM114A1	−0.57	−0.62
**siSF-1 upregulated**		
MYB	0.98	0.47
TGFB2	0.76	0.76
GJB2	0.76	0.74
TFPI2	0.74	0.74
CD164	0.73	0.41
RNFT1	0.72	0.49
GPR37	2.72	−0.51
GPR64	1.90	−0.86
CHGB	1.88	−0.53
ANKFN1	1.83	−1.32
DKK2	1.48	−0.65
FGF13	1.32	−0.87
PIP4K2A	1.28	−0.42
APCDD1	1.24	−0.85
SLC17A5	1.22	−0.44
STC1	0.96	−0.90
FST	0.94	−0.80
FAH	0.87	−0.51
LIN7B	0.85	−0.62
ANGPTL2	0.85	−0.65
GRN	0.82	−0.48
OSBPL6	0.82	−0.52
SLC1A1	0.82	−0.64
SMAD9	0.81	−0.60
ASAP1	0.78	−0.47
LRP12	0.77	−0.63
CTTNBP2	0.74	−0.49
DACH2	0.72	−0.73
KCTD12	0.70	−0.80
ANXA2	0.68	−0.50
ZNF462	0.67	−0.53
SLC20A2	0.62	−0.47
MUM1L1	0.61	−0.42
ANXA2P1	0.61	−0.48
ACPL2	0.59	−0.82

Values given in log_2_ (fold change).

Tables of the 35 most up- and downregulated genes, respectively, in the siRNF31 arrays can be found in the supplementary materials (Tables S7, 8, 9, 10).

In the Control vs. siRNF31 microarray experiment, the known RNF31 target genes StAR and CYP19A1 [14] were found to be regulated. The microarray also identified, and qPCR confirmed, MC2R, SCARB1, SULT2A1 and CYP17A1 as putative targets for RNF31. As all of these genes are reported SF-1 targets this fits our model wherein the corepressor complex including RNF31 is recruited by SF-1 to its target promoters. Future work has to confirm this model for these new putative target genes, for example using chromatin immunoprecipitation (ChIP) assays.

In general, knockdown of SF-1 or RNF31 does not seem to fully inhibit cAMP induced gene regulation, even though it attenuates the response as the basal level changes, and thus the induction starts from a higher (RNF31) or lower (SF-1) level. This confirms the finding of Sugawara *et al.* [34] that SF-1 is not the major factor for cAMP induction of steroidogenic enzymes. Part of the remaining response, however, is likely due to traces of SF-1 and RNF31 still being expressed, as the siRNA does not completely abolish their expression. A Venn diagram showing the overlap between the cAMP-treated samples can be found in Figure 3D.

We used pathway-enrichment analysis to mine the large data sets for functional implications (Table 3). Interestingly, the top five most enriched processes affected when RNF31 was silenced are all indicative of steroidogenic processes. This supports that RNF31 is indeed involved in steroidogenic regulation, in line with our proposed model of RNF31 action. Additional sub-network enrichment analysis revealed that DAX-1 is statistically linked (p = 0.0015) to the set of differentially regulated genes affected by silencing of RNF31. DAX-1 turns up as a hub among the genes related to steroidogenesis that were upregulated upon RNF31 knockdown (Figure 2C). This strengthens our hypothesis that RNF31 works together with DAX-1 in regulating a subset of the SF-1 target genes.

**Table 3:** Top 20 GO biological processes in functional analysis of siRNF31 microarray results.

Biological process	Count	P-Value
GO:0006694∼steroid biosynthetic process	12	6.75E-07
GO:0008202∼steroid metabolic process	14	1.52E-04
GO:0008610∼lipid biosynthetic process	18	1.82E-04
GO:0016126∼sterol biosynthetic process	6	5.20E-04
GO:0016125∼sterol metabolic process	9	7.42E-04
GO:0030198∼extracellular matrix organization	9	9.00E-04
GO:0030199∼collagen fibril organization	5	2.20E-03
GO:0046394∼carboxylic acid biosynthetic process	10	3.14E-03
GO:0016053∼organic acid biosynthetic process	10	3.14E-03
GO:0043062∼extracellular structure organization	10	4.38E-03
GO:0008203∼cholesterol metabolic process	7	8.72E-03
GO:0042471∼ear morphogenesis	6	8.83E-03
GO:0043583∼ear development	7	1.01E-02
GO:0009719∼response to endogenous stimulus	16	1.22E-02
GO:0007267∼cell-cell signaling	21	1.25E-02
GO:0044092∼negative regulation of molecular function	14	1.30E-02
GO:0006695∼cholesterol biosynthetic process	4	1.33E-02
GO:0016055∼Wnt receptor signaling pathway	8	1.46E-02
GO:0045859∼regulation of protein kinase activity	14	1.66E-02
GO:0009064∼glutamine family amino acid metabolic process	5	1.78E-02

As mentioned, 9 of 50 genes were upregulated by siRNF31 and downregulated by siSF-1. However, the majority of the genes, 29 genes, react the opposite way; they are upregulated by siSF-1 and downregulated by siRNF31. Remaining 12 genes are regulated in the same direction by both treatments. How the latter groups of genes are regulated by SF-1 and RNF31 at a mechanistic level and if this involves an SF-1/RNF31 interaction remains to be investigated. The 29 genes could for example be the result of an indirect regulation where SF-1 upregulates a repressor.

### SF-1 and RNF31 affect genes involved in Wnt/β-catenin signaling

We performed pathway-enrichment analysis on the siSF-1 dataset (selected gene ontology (GO) terms in Table 4, first 50 GO terms in Table S11) and found several interesting regulated pathways, among them the Wnt/β-catenin pathway. Activation of the Wnt signaling via binding of Wnt proteins to the Frizzled receptors inhibits phosphorylation of β-catenin by Casein-dependent kinase 1 and Glycogen synthase kinase-3; this leads to β-catenin stabilization and relocalization to the nucleus, where it can activate TCF-dependent transcription of target genes including Axin-2 and Cyclin D. SF-1 and Wnt/β-catenin signaling have previously been shown to synergize in the activation of SF-1 target genes such as Inhibin α [35] and luteinizing hormone [36] in the gonadal tissues, probably through direct interaction between β-catenin and SF-1, something that has also been shown for LRH-1 [37], SF-1's closest relative within the NR family.

**Table 4:** Selected GO biological processes from siSF-1 pathway-enrichment analysis.

Term	Count	P-Value
2. Positive regulation of developmental process	16	9.15E-05
4. Positive regulation of macromolecule biosynthetic process	25	3.69E-04
5. Adherens junction	11	4.61E-04
6. Wnt receptor signaling pathway	10	4.74E-04
7. Positive regulation of cell differentiation	13	5.98E-04
8. Response to hormone stimulus	17	6.01E-04
9. Steroidogenesis	4	6.21E-04
10. Response to organic substance	26	6.38E-04
11. Osteoblast differentiation	6	7.20E-04
12. 3′,5′-cyclic-nucleotide phosphodiesterase activity	5	7.35E-04
13. Positive regulation of macromolecule metabolic process	29	7.65E-04
19. Sex differentiation	10	1.11E-03
27. Regulation of cell proliferation	26	2.16E-03
45. TGF-beta signaling pathway	7	4.16E-03

**Figure 4:**
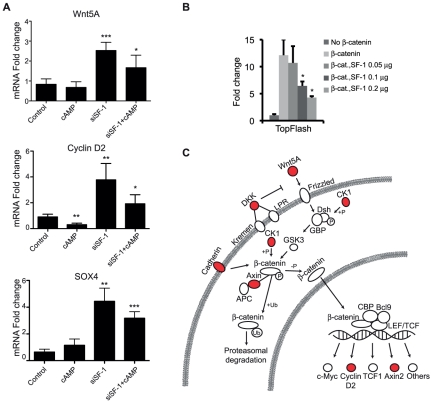
Putative SF-1 targets in the Wnt/β-catenin-signaling pathway. (**A**) qPCR data of three genes in the Wnt/β-catenin pathway that are changed after siSF-1 knockdown. (**B**) Change in β-catenin responsive reporter plasmid TopFlash activity with increasing amounts of SF-1. Values shown are fold change compared to reporter plasmid luciferase activity without transfected β-catenin. (**C**) Schematic Wnt/β-catenin pathway with genes changed by siSF-1 marked in red (upregulation). Error bars show standard deviation. Statistical analysis done with Student's t-test (* p<0.05; **p<0.01;***p<0.001).

Our analyses show that genes involved in the Wnt/β-catenin signaling pathway is overrepresented among both SF-1- and RNF31-regulated genes (Figure 4B). Transcripts for the Wnt protein Wnt5a were detected as SF-1 regulated by both microarray and qPCR (Figure 4A), as was the activator CK-1γ, the repressors DKK2 and AXIN2 and the Wnt target genes Cyclin D2 and AXIN2. The genes are mostly upregulated in response to SF-1 knockdown, identifying SF-1 as a functional repressor of their transcription. The mechanistic explanation of this remains to be investigated but possibilities include transrepression on the promoters or decreased expression of another transcriptional repressor. Figure 4C shows the placement of the siSF-1 regulated genes within the Wnt/β-catenin pathway.

Using the Wnt/β-catenin luciferase reporter plasmid TopFlash, we could see that increasing concentrations of SF-1 repressed β-catenin induced transcription (Figure 4B). Thus it seems that SF-1 can repress Wnt/β-catenin signaling directly which might explain the increase of Wnt/β-catenin targets Cyclin D2 and Axin 2 upon SF-1 knock down in NCI-H295R cells.

For RNF31, GO term-enrichment analysis identified the Wnt pathway as significantly affected (p = 0.015) and 8 Wnt-pathway targets were found among the differentially expressed genes (DKK2, DKK1, NXN, KREMEN2, NLK, MITF, PPM1A, FZD6). Of these, only DKK2 was also changed by siSF-1. All genes but NLK were downregulated by RNF31 knockdown, indicating that RNF31 is directly or indirectly repressing these genes.

Interestingly, SF-1 and Wnt/β-catenin signaling are functionally connected both in the development and homeostasis of the adrenal cortex. Conditional knockout mice where β-catenin is removed from SF-1-expressing cells show adrenal agenesis, similar to the SF-1 knockout mice [38]. Development initiates normally but the KO adrenals show increased differences in size compared to wild type and at E18.5 no traces of the developing gland can be seen [38]. In addition, Wnt4 KO mice show gonadal developmental defects with adrenal-type cells infiltrating the gonads indicating that Wnt ligands play a role in directing the cellular fate of the adrenogonadal primordium [39]. In the adult, active β-catenin signaling is restrictively located to the subcapsular region of the adrenal cortex [38]. This region is of special interest, as it is believed to harbor the progenitor cells responsible for the regenerative capacity of the adrenal cortex. These cells are also SF-1 and DAX-1 positive and may stem from the fetal cortical zone (see [1] for a recent review). A conditional KO where β-catenin signaling is impaired but not totally ablated in the SF-1 expressing cells has been made [38]. Interestingly, these mice are born with functioning adrenal glands but the cortex degenerates with age, indicating that the subcapsular progenitors are not functioning properly or that there is a shortage of progenitor cells, again highlighting the importance of β-catenin signaling in the SF-1 positive progenitors.

Aberrant β-catenin signaling is also a common feature in cancer, including somatic, activating mutations of β-catenin, which lead to continuous activity of β-catenin target genes. Such mutations have been identified both directly in adrenal tumors and in the cell line NCI-H295R. In NCI-H295R the S45P mutation has been shown to cause the β-catenin pathway to be constitutively active [40]. The Wnt target Cyclin D2 [41] that is upregulated in our data is important for cell-cycle progression and increase in its expression might increase tumor growth.

A thorough mapping of the functional and mechanistic relationship between the SF-1 and Wnt/β-catenin pathways promises to provide insights into both adrenal development and homeostasis that could eventually lead to efficient strategies for fighting adrenocortical carcinomas. Especially encouraging in this aspect is the data showing that repression of either pathway inhibits the growth of adrenocortical cells [42], [43]. Our data show that SF-1 may actively impact Wnt/β-catenin signaling both directly by inhibiting β-catenin-induced transcription (Figure 4 B) and by affecting the levels of proteins involved in the Wnt/β-catenin pathway.

### SF-1 knockdown regulates genes involved in TGFβ signaling

**Figure 5:**
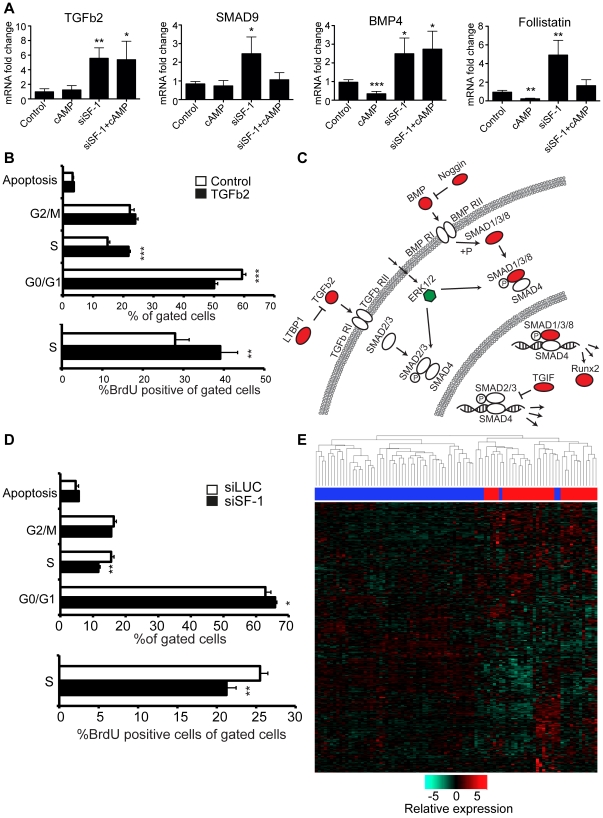
Putative SF-1 target genes in TGFβ signalling pathways identified from pathway enrichment analysis in the DAVID online resource, siSF-1 effect on H295R cell proliferation. (**A**) qPCR showing the effect on mRNA level of SF-1 knockdown ± cAMP treatment proteins in the TGFβ-pathway. (**B**) Flow-cytometry analysis of cells treated with TGFβ2 show significantly more cells in S-phase and less in G1/G0 after TGFβ2 treatment. (**C**) Schematic representation of the TGFβ-pathway with genes changed by SF-1 knockdown marked red for upregulation and green for downregulation. (**D**) Flow cytometry analysis of siSF-1 or control treated cells show more cells in G1/G0-phase and less in S-phase after SF-1 knockdown. (**E**) The SF-1 gene expression profile can classify malignant from benign adrenocortical tumors in clinical samples. The heatmap shows relative expression of genes differentially expressed in our siSF-1 microarray experiments in a study cohort of 58 ACCs and 34 ACAs [53]. Up- and downregulation is indicated by red and green, respectively, scaled across rows. The top bar illustrates the significant (p<0.01) separation of carcinomas (red) from adenomas (blue) by hierarchical clustering. Error bars show standard deviation. Statistical analysis done with Student's t-test (* p<0.05; **p<0.01; ***p<0.001).

Overrepresentation analysis identified TGFβ signaling as a significantly enriched pathway (p = 0.004) among SF-1-regulated genes. Nine genes classified as belonging to the TGFβ pathway were regulated (TGFB2, BMP4, SMAD9, MAPK1, NOG, LTBP1, FST, TGIF1, RUNX2) by SF-1 knockdown. We selected four of these (TGFB2, SMAD9, FST and BMP4) and confirmed their regulation using qPCR (Figure 5A). We further mapped the locations of most of the regulated genes in the TGFβ pathway (Figure 5C). Prominently, all TGFβ-connected genes but MAPK1 (ERK2) were upregulated upon SF-1 knockdown. This indicates a role for SF-1 as a negative regulator, direct or indirect, of the expression of genes in the TGFβ pathway. The functional output of these changes awaits future investigations.

TGFβ signaling has previously been shown to act inhibitive on steroidogenesis in the NCI-H295R cell line [44]. A connection to SF-1 was made when it was shown that TGFβ1 downregulates SF-1 expression [45] and SMAD3 inhibits SF-1-induced CYP17 expression [46], but the molecular mechanisms and physiological consequences remain unclear.

The TGFβ family of cytokines contains over 30 members in humans and drives processes that promote differentiation and control proliferation in pre-malignant tissues. However, in some instances the dysregulation occurring in malignant tumors can turn TGFβ cytokines into mitogens that stimulate continuous growth and metastasis [47]. To investigate the effects of TGFβ2, which was upregulated by SF-1 knockdown, in adrenocortical NCI-H295R cells, we treated the cells with TGFβ2 and measured cell proliferation 16 h later using flow cytometry. The number of cells in S-phase increased and those in G1/G0 decreased upon treatment, indicating that the rate of proliferation increased (Figure 5B). Thus, the NCI-H295R cells have acquired the deregulated TGFβ signaling of a cancer cell. It has been suggested that childhood adrenocortical tumors could be treated by lowering the expression levels of SF-1 or by inhibiting its transcriptional activities using antagonist ligands. Potentially, the increased TGFβ2 expression resulting from SF-1 knockdown could pose a problem for such a therapy if childhood tumors have dysregulated TGFβ signaling like the NCI-H295R cells, and this needs to be explored further.

### SF-1 and genes involved in adrenocortical development and cancer

As described above, we found genes involved in Wnt- and TGFβ-signaling pathways to be enriched among the SF-1-regulated genes, as were genes related to developmental processes and cellular proliferation. SF-1 has been linked to adrenocortical cancer in general and to childhood adrenocortical tumors (ACTs) in particular [13]. Studies have shown that SF-1 is overexpressed in childhood ACTs compared to normal adrenal tissue [4], [5] and that in NCI-H295R cells overexpression of SF-1 leads to increased proliferation and tumor growth [48]. In a paper studying the genome–wide effects of SF-1 overexpression in NCI-H295R cells, Doghman *et al.* showed that FATE1 is involved in mediating this effect but the exact mechanism remains elusive [48]. We compared the two datasets: ours, where we have knocked down SF-1 and theirs, where they have overexpressed SF-1, and we found an overlap of 12 genes out of 71 genes that are comparable between the different microarray platforms (Doghmann *et al.* report a total of 98 genes as regulated by overexpression). 10 of the 12 genes are regulated in the opposite direction in the knockdown compared to the overexpression: FATE1, C21orf25, KIAA1913, APOA1, HSPB7 and SULT2A1 are upregulated by SF-1 overexpression and downregulated by SF-1 knockdown while ANKFN1, ENPP2, FGF13 and ACPL2 are regulated the opposite way. The low degree of overlap could be due to the effects of clonal selection when Doghman *et al.* made the SF-1 overexpressing subclone of the NCI-H295R cells or other differences in experimental setup. Surprisingly, the overexpression data set does not show changes in known SF-1 target genes such as StAR or CYP17A1. This could indicate that the NCI-H295R cells already contain maximum dosage of SF-1 for full SF-1 activity or that some activating signal or ligand is functioning inappropriately when SF-1 is overexpressed. The Doghman study also showed that RNAi-mediated SF-1 knockdown decreased proliferation in NCI-H295R cells. We also saw a slight reduction of proliferation rate in siSF-1-treated cells compared to control-treated cells (Figure 5D). The reduction of cells in S-phase was statistically significant at the 5%-level (both from histogram and BrdU staining).

We found that Insulin-like growth factor-II (IGFII) gene IGF2 was downregulated following SF-1 knockdown. IGF2 encodes a protein known to have proliferative effects on adrenal cells [2]. It is abundantly expressed during fetal development and thought to govern growth of the fetal zone of the adrenal cortex. After birth, levels of IGFII drop and the fetal zone regresses in an apoptotic process while the adult zonation of the cortex takes place. It has also been shown that IGFII affects steroidogenesis in the adrenal. IGF2 is overexpressed in most adrenocortical carcinomas and its overexpression is believed to play a pivotal role in the transformation of a tumor from a benign to a malignant state [2]. The NCI-H295R cell, which is derived from a malignant adrenocortical carcinoma, has been shown to produce IGF2, which has a proliferative effect on the cells [49]. IGF2 downregulation upon SF-1 knockdown could be beneficial with respect to decreased tumor growth and of interest therapeutically as different treatments designed to decrease IGF2 levels have been suggested for adrenocortical carcinoma [1]. Interestingly, cAMP treatment of the cells did not affect IGF2 mRNA levels indicating that the effect may be due to SF-1 activity directly and not due to decreased hormone levels in the growth medium.

During embryonic development, SF-1 expression has been shown to be driven by a fetal adrenal enhancer element (FAdE) located within intron 4. This enhancer element contains binding sites for the homeobox protein Pre-B-Cell Leukemia Transcription Factor-1 (PBX1) and SF-1 itself. PBX1 seems to initiate SF-1 expression in early development and SF-1 then drives its own expression [50]. In line with this data is the observation that PBX1 is expressed in the developing adrenal and that PBX1 knockout mice (embryonically lethal at day 15/16) suffer from adrenal agenesis [51]. PBX1 also seems to be important for adult adrenal proliferation as PBX1 haploinsufficient mice (PBX1^+/−^) have lower adrenal weight and a reduced regenerative capacity of the adrenal than wild type mice [52]. Interestingly, PBX1 and SF-1 seem to share steroidogenic target genes within the adrenal (MC2R and CYP17) and SF-1 has been reported to upregulate PBX1 expression in NCI-H295R cells in transfection assays [52]. In our data however, loss of SF-1 seems to increase PBX1 expression, perhaps via some compensatory mechanism. Increased PBX1 expression could be of importance for adrenal proliferation rate and this should be investigated if SF-1 antagonists are tried for reducing adrenal tumor growth.

Our findings were made in the NCI-H295R cell line, the standard adrenal cell line expressing SF-1 that is highly relevant for the human adrenal cortex. As this represents the only human cell line available to study the effects of SF-1 in the human adrenal cortex and in adrenocortical carcinoma, we cannot replicate these data in another cell line. To address this limitation, and to assess the clinical relevance of the genes affected by SF-1 knockdown in NCI-H295R, we interrogated their expression in a recent transcriptome-profiling study of 92 adrenocortical tumors [53]. By hierarchical clustering, our gene set (Control vs. siSF-1) was able to part the tumor samples into two major clusters, whereof one consisted of all (34/34) malignant ACCs and six (6/58) adrenocortical adenomas (ACAs). The other cluster consisted entirely of benign ACAs (52/58) (Figure 5E). The clustering of ACAs showed statistical significance per bootstrap resampling for a majority of the samples (p<0.05, data not shown). Although the clustering of ACCs did not show statistical significance per bootstrap resampling, the association of tumor type with cluster was significant (p<0.01), as determined by Fisher's exact test. The regulation of these genes did not completely concur with up-/downregulation in our microarray experiments; nevertheless, it shows that they are involved in the tumorigenic processes in aggressive adrenocortical tumors and it is also possible that there is a subtype of ACCs where SF-1 signaling is aberrant. The strong ability of the SF-1 profile to classify between benign and malignant clinical samples of adrenocortical tumors indicates SF-1 signalling as imperative during development of adrenocortical cancer.

### Conclusions and future directions

In this study we have mapped the effects of depleting adrenocortical cells of either SF-1 or RNF31. We found a strong connection between RNF31 and the steroidogenic pathway, which corroborates our previous result connecting SF-1, steroidogenesis and RNF31. Our data additionally suggest a role for SF-1 beyond regulation of steroid hormone production in the adrenal. Connections to TGFβ and Wnt/β-catenin signaling are made, for the first time indicating that SF-1, through yet to be described mechanisms, may have a repressive effect on these intracellular signaling pathways.

**Figure 6:**
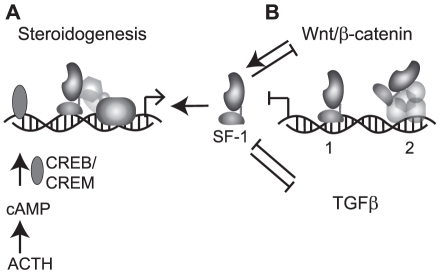
Hypothesis of SF-1 mechanism acting both *in cis* and *in trans*. (**A**) Classical SF-1 action on steroidogenic enzyme gene promoters. SF-1 binds promoters *in cis* and recruit coactivators and the general transcription machinery to activate transcription. Raised intracellular cAMP levels due to ACTH (in the adrenal) activation of MC2R activates the CREB/CREM transcription factors that work synergistically to further increase transcription rates. Input from Wnt/β-catenin signalling through direct binding of β-catenin to SF-1 can also increase transcription. (**B**) Possible mechanisms of SF-1 dependent repression of Wnt/β-catenin signaling. 1. SF-1 binds *in cis* to promoters but due to post-translational modifications and/or specific corepressor recruitment represses instead of activates target gene transcription. 2. SF-1 binds *in trans* to transcription factor complex and directs corepressors to the site to repress transcription. This could also be mediated by post-translational modifications like SUMOylation. A third option is that transcription factors or corepressors whose expression is activated by SF-1 acts as repressors of the TGFβ and Wnt/β-catenin signalling making SF-1 an indirect regulator.

Transrepression is a specific mechanism of transcriptional crosstalk that allows some nuclear receptors to inhibit other signaling pathways [54]. Intriguingly, SF-1's closest homologue LRH-1 (NR5A2) can transrepress genes involved in the hepatic immune response via a tethering mechanism [55]. LRH-1 and SF-1 share many functional similarities and it will be interesting to see if SF-1 mimics the tethering mechanisms of LRH-1. In the case of LRH-1, SUMOylation appears to play a pivotal role in the transrepression mechanism by allowing it to tether to target transcription factor-corepressor complexes. SUMOylation is also known to repress the actions of SF-1 in part by decreasing its affinity to certain DNA response elements [25]. Perhaps this, in combination with an LRH-1-like tethering mechanism, could trigger SF-1 transrepression of pathways such as TGFβ or Wnt (Figure 6). Experiments exploring such hypotheses will hopefully lead to advances in the SF-1 field and also result in a better understanding of the physiology of SF-1 in its target tissues.

## Materials and Methods

### Cell lines and cell culture

The human adrenocortical carcinoma cell line NCI-H295R was acquired from ATCC (CRL-2128). Cells were maintained in DMEM/F12 (Invitrogen) medium supplemented with 1% ITS+ (BA Biosciences) and 2.5% NU-Serum (BA Biosciences) and subcultured every 2–3 days.

### siRNA and forskolin treatment

A mixture of four siRNA oligos was used for knockdown of SF-1 (siGENOME SMARTpool M-003429, Dharmacon, Thermo Scientific) and RNF31 (siGENOME SMARTpool D-021419, Dharmacon, Thermo Scientific), respectively. As non-targeting control an oligo targeting luciferase was used (D-001206-14, Dharmacon, Thermo Scientific). siRNA transfections were performed as described in [14]; for qPCR 15 mm cell culture dishes were used; for microarrays 10 cm dishes. 25 µM forskolin was added to indicated samples 16 h before harvest.

### RNA isolation and qPCR

RNA was harvested using the E.Z.N.A. total RNA kit (Omega Biosciences) according to the manufacturer's instructions. RNA concentration was measured and quality assessed with a Nanodrop (ND-100) spectrophotometer. cDNA (500 ng RNA/reaction) synthesis was performed using Superscript III (Invitrogen) according to Invitrogen's protocol. qPCR primers were designed using Primer Express (Applied Biosciences) or Primer-BLAST (NIH) and span exon-exon junctions in all possible cases. qPCR was run on 7500 Fast instruments (Applied Biosciences) using Fast SYBR Green Master Mix (Applied Biosciences), qPCR data were analyzed using the ΔΔC_T_-method. All samples were run in biological quintuplicate (n = 5) and analyzed for statistical significance compared to the control sample using Student's t-test with Welch's correction (not assuming equal variance). A confidence interval of 95% was used. Calculations were performed in GraphPad Prism 5.0 for Macintosh.

### Protein preparation and Western blot

Cells were lysed in 200 µl RIPA buffer (1% NP-40, 0.1% Triton X-100, 150 mM NaCl, 50 mM Tris, Complete protease inhibitors (Roche), pH 7.4). Protein concentration was assayed using the BCA kit (Pierce). Equal amounts of protein were mixed with SDS-PAGE sample buffer, boiled and run on 10% SDS-PAGE gel. Proteins were transferred to Hybond-C super membrane (GE Healthcare), membranes were blocked with 5% milk in PBS +0.05% Tween-20 (PBS-T) and incubated with antibody (αStAR (rabbit polyclonal, Affinity Bioreagents) dilution 1∶1,000, αSF-1 (rabbit polyclonal, Upstate) dilution 1∶1,000, αRNF31 (rabbit polyclonal, in-house [14]), dilution 1∶5,000 or αβ-actin (mouse monoclonal, Sigma Aldrich) dilution 1∶10,000) for 2 h at room temperature or 4°C overnight. Membranes were washed in PBS-T and incubated with α-rabbit/mouse-HRP-coupled antibody (GE Healthcare), 1∶10,000 dilution, for 1 h and washed 3 times in PBS-T. SuperSignal West Pico chemiluminescent substrate (Pierce) was added according to manufacturer's instructions and blots developed on light-sensitive film (GE Healthcare).

### Microarrays and bioinformatics

Microarray analysis was essentially performed as described in [56]. In short, RNA was hybridized to Operon's nucleotide arrays from Microarrays Inc. Obtained TIFF-images were analyzed in GenePix Pro 6.1 where they were manually inspected for irregularities. Data were then further analyzed in the R statistical environment using the packages Bioconductor bundle [57], Limma [58], Aroma and KTH. Unreliable spots were filtered and intensity data was normalized using the print-tip lowess method within the Aroma package. Afterwards, a linear model using the least-square method fit for each gene and the empirical Bayes moderated t-test within the Limma package was applied to the data, which generated a list of differentially expressed genes with p-values and B scores. Genes not detected in at least three out of four arrays were discarded. Raw data and detailed protocols were submitted in accordance with MIAME guidelines and are available from the ArrayExpress data repository using the accession number E-MEXP-3259.

Microarray data were confirmed by qPCR analysis of 20 genes in quintuplicate samples, where all samples were independent of the microarray samples.

The online DAVID Bioinformatics resource v 6.7 [59], [60] was then used for analysis of overrepresented gene ontology (GO) groups by biological process and involved pathways. P-values in DAVID below 0.05 were considered significant.

Pathway Studio analysis software (Ariadne Genomics) was used for sub-network enrichment analysis (SNEA).

Data from de Reyniès *et al*. [53] was downloaded from ArrayExpress, accession E-TABM-311. Expression values for genes of interest were extracted with gene symbol as identifier, for duplicate probes the mean was used. A heatmap was generated by hierarchical clustering of rows and columns using average linkage with Pearson's correlation as similarity metric. All analysis was performed in R using the packages *gplots* and *pvclust* for statistical analysis. Certainty of clusters was determined by multiscale bootstrap resampling. Significance of association between clustering and tumor type was assessed by two-sided Fisher's exact test.

### Promoter assay

Promoter assays were performed essentially as described in [14]. 0.5 µg of either pGL3-Basic, pGL3-Basic-SUL2A1-LUC or pGL3-Basic-DAX-1-LUC reporter plasmid together with 0.1 µg β-Galactosidase reporter plasmid was used.

Luciferase readings were normalized against β-gal measurements. Mean fold-change of forskolin (cAMP) compared to mock-treated cells and standard deviations were calculated and statistical significance tested using two-tailed, unpaired, Student's t-test.

The Wnt/β-catenin reporter TopFlash was transfected to HeLa cells together with β-catenin and increasing amounts of SF-1. Luciferase activity was read after 24 h and fold change compared to TopFlash without β-catenin was caluclated. Statistical significance was tested with two-tailed, unpaired Student's t-test.

### Proliferation assay

Cells were seeded and, when indicated, transfected with siRNA as described in *siRNA and qPCR*. 5nM TGFβII was added to indicated samples 24 h after seeding and cells were then treated for 16 h. At 15 h 30 µM BrdU was added to each sample to label cells in S-phase. Cells were harvested by trypsination, pelleted by centrifugation, fixed in 1 ml ice-cold 70% ethanol and stored at −20°C. On the day of analysis, cells were labeled with anti-BrdU-antibody (BD Biosciences) and propidium iodine and assayed using flow cytometry. Mean values and standard deviations of five replicates are presented and statistical significance tested as in the promoter and qPCR assays.

## Supporting Information

Table S1 **35 most upregulated genes in SF-1 RNAi-treated cells.**
(PDF)

Table S2 **35 most downregulated genes in SF-1 RNAi-treated cells.**
(PDF)

Table S3 **35 most upregugulated genes in SF-1 RNAi+cAMP-treated cells.**
(PDF)

Table S4 **35 most downregugulated genes in SF-1 RNAi+cAMP-treated cells.**
(PDF)

Table S5 **35 most upregulated genes in cAMP-treated cells.**
(PDF)

Table S6 **35 most downregulated genes in cAMP-treated cells.**
(PDF)

Table S7 **35 most upregulated genes in RNF31 RNAi-treated cells.**
(PDF)

Table S8 **35 most downregulated genes in RNF31 RNAi-treated cells.**
(PDF)

Table S9 **35 most upregugulated genes inRNF31 RNAi+cAMP treated cells.**
(PDF)

Table S10 **35 most downregugulated genes inRNF31 RNAi+cAMP treated cells.**
(PDF)

Table S11 **50 first hits in DAVID functional annotation chart of siSF-1 microarray.**
(PDF)
