# Supplementary material for: Knockdown of SF-1 and RNF31 Affects Components of Steroidogenesis, TGFβ, and Wnt/β-catenin Signaling in Adrenocortical Carcinoma Cells
Source: PLoS One. 2012 Mar 9;7(3):e32080. doi: 10.1371/journal.pone.0032080 (PMC3302881; doi:10.1371/journal.pone.0032080)
Supplement: Table S1 — 35 most upregulated genes in SF-1 RNAi-treated cells. (PDF) [file pone.0032080.s001.pdf]

Supplementary table 1. 35 most upregulated genes in SF-1 RNAi-treated cells

Gene Symbol	Description	Fold Change
GPR37	Endothelin B receptor-like protein 1 (ETBR-LP-1) Parkin-associated endothelin receptor-like receptor (PAELR)	6.60
GNG2	Guanine nucleotide-binding protein G(I)/G(S)/G(O) gamma-2 subunit precursor (G gamma-I).	5.04
NELL2	Protein kinase C-binding protein NELL2 precursor (NEL-like protein 2) (Nel-related protein 2).	4.01
GPR64	Epididymis-specific protein 6 (He6 receptor)	3.74
CHGB	Secretogranin-1 precursor (Secretogranin I) (SgI) (Chromogranin B) (CgB) Contains: GAWK peptide; CCB peptide	3.68
ANKFN1	Ankyrin repeat and fibronectin type-III domain-containing protein 1	3.55
GLCE	D-glucuronyl C5-epimerase (EC 5.1.3.-) (Heparin/heparan sulfate:glucuronic acid C5 epimerase)	3.29
NKD1	Naked cuticle homolog 1	2.97
EMB	Embigin precursor	2.96
DKK2	Dickkopf-related protein 2 precursor (Dkk-2) (Dickkopf-2) (hDkk-2)	2.78
CXADR	Coxsackievirus and adenovirus receptor precursor (Coxsackievirus B- adenovirus receptor) (hCAR)	2.72
GAP43	Neuromodulin (Axonal membrane protein GAP-43) (Growth-associated protein 43) (PP46) (	2.69
PCDH18	Protocadherin-18 precursor	2.66
NEO1	Neogenin precursor	2.60
SLC7A8	Large neutral amino acids transporter small subunit 2 (L-type amino acid transporter 2) (hLAT2)	2.59
CCND2	G1/S-specific cyclin-D2	2.57
FGF13	Fibroblast growth factor 13 (FGF-13) (Fibroblast growth factor homologous factor 2) (FHF-2)	2.50
CSPG2	Versican core protein precursor (Large fibroblast proteoglycan) (Chondroitin sulfate proteoglycan core protein 2)	2.49
VASH2	Vasohibin-2 (Vasohibin-like protein)	2.48
ST8SIA4	CMP-N-acetylneuraminate-poly-alpha-2,8-sialyltransferase	2.48
ANXA1	Annexin A1 (Annexin I) (Lipocortin I)(Phospholipase A2 inhibitory protein)	2.48
ATP1B3	Sodium/potassium-transporting ATPase subunit beta-3	2.45
MATN2	Matrilin-2 precursor	2.44
PIP5K2A	Phosphatidylinositol-4-phosphate 5-kinase type-2 alpha (EC 2.7.1.68)	2.43
ACPP	Prostatic acid phosphatase precursor (EC 3.1.3.2)	2.41
FGF13	Fibroblast growth factor 13 (FGF-13) (Fibroblast growth factor homologous factor 2) (FHF-2)	2.40
LRRC49	Leucine-rich repeat-containing protein 49 (Tubulin polyglutamylase complex subunit 4) (PGs4)	2.37
APCDD1	Protein APCDD1 precursor (Adenomatosis polyposis coli down-regulated 1 protein)	2.36
SLC17A5	Sialin (Solute carrier family 17 member 5) (Sodium/sialic acid cotransporter) (AST) (Membrane glycoprotein HP59)	2.33
KCNJ8	ATP-sensitive inward rectifier potassium channel 8	2.32
CCDC74B	Coiled-coil domain containing 74B	2.27
FKBP5	FK506-binding protein 5 (EC 5.2.1.8) (Peptidyl-prolyl cis-trans isomerase)	2.25
PARP8	Poly [ADP-ribose] polymerase 8 (EC 2.4.2.30) (PARP-8)	2.24
MYO6	Myosin-6 (Myosin VI) (Unconventional myosin VI)	2.20
ELL2	RNA polymerase II elongation factor ELL2	2.19
