# Supplementary material for: Knockdown of SF-1 and RNF31 Affects Components of Steroidogenesis, TGFβ, and Wnt/β-catenin Signaling in Adrenocortical Carcinoma Cells
Source: PLoS One. 2012 Mar 9;7(3):e32080. doi: 10.1371/journal.pone.0032080 (PMC3302881; doi:10.1371/journal.pone.0032080)
Supplement: Table S2 — 35 most downregulated genes in SF-1 RNAi-treated cells. (PDF) [file pone.0032080.s002.pdf]

Supplementary table 2. 35 most downregulated genes in SF-1 RNAi-treated cells

Gene Symbol	Description	Fold Change
CYP11A1	Cytochrome P450 11A1 (Cholesterol side-chain cleavage enzyme) P450(scc)	0.29
NR5A1	nuclear receptor subfamily 5, group A, member 1 (NR5A1)	0.34
STAR	Steroidogenic acute regulatory protein, mitochondrial precursor (StAR) (StARD1)	0.36
TMEM200A	KIAA1913, Transmembrane protein 200A	0.39
TFRC	Transferrin receptor protein 1 (TfR1) (CD71 antigen) (T9) (p90)	0.41
KCNK3	Potassium channel subfamily K member 3 (Acid-sensitive potassium channel protein TASK-1) (TWIK-related acid-	0.41
SIGLEC11	Sialic acid-binding Ig-like lectin 11 precursor (Siglec-11) (Sialic acid-binding lectin 11).	0.43
RORB	RORB	0.45
MAPK1	Mitogen-activated protein kinase 1 (EC 2.7.11.24) (Extracellular signal-regulated kinase 2) (ERK-2) (Mitogen-activa	0.45
RHOB	Rho-related GTP-binding protein RhoB precursor (H6)	0.47
CYP17A1	Cytochrome P450 17A1 (EC 1.14.99.9) (CYPXVII) (P450-C17) (P450c17) (Steroid 17-alpha-monooxygenase) (Ste	0.47
GALC	Galactocerebrosidase precursor (EC 3.2.1.46) (GALCERase) (Galactosylceramidase) (Galactosylceramide beta-gala	0.49
HIST1H2BJ	Histone H2B type 1-J (H2B.r) (H2B/r) (H2B.1)	0.50
CSN1S1	Alpha-S1-casein precursor [Contains: Casoxin-D]	0.50
Q86V52	-	0.50
FHL2	Four and a half LIM domains protein 2 (FHL-2) (Skeletal muscle LIM- protein 3) (SLIM 3) (LIM domain protein D	0.51
LSP1	Lymphocyte-specific protein 1	0.52
VSNL1	Visinin-like protein 1 (VILIP) (Hippocalcin-like protein 3) (HLP3)	0.52
IGDCC4	immunoglobulin superfamily, DCC subclass, member 4	0.52
IGF2	Insulin-like growth factor II precursor (IGF-II) (Somatomedin A) [Contains: Insulin-like growth factor II Ala-25 Del	0.52
TNS3	Tensin-like SH2 domain containing 1	0.53
SCARB1	Scavenger receptor class B member 1 (SRB1) (SR-BI) (CD36 antigen-like 1) (CD36 and LIMPII analogous 1) (CLA	0.53
DNAH11	Ciliary dynein heavy chain 11 (Axonemal beta dynein heavy chain 11).	0.54
FBXW10	FBXW10 protein	0.54
MGC13053	Hypothetical MGC13053	0.54
EEF1AL3	Eukaryotic translation elongation factor 1 alpha 1 (Fragment)	0.54
FKBP4	FK506-binding protein 4 (EC 5.2.1.8) (Peptidyl-prolyl cis-trans isomerase) (PPIase) (Rotamase) (p59 protein) (HSP-	0.55
ZBTB7C	zinc finger and BTB domain containing 7C	0.55
ITGA9	Integrin alpha-9 precursor (Integrin alpha-RLC)	0.55
PDE2A	cGMP-dependent 3',5'-cyclic phosphodiesterase (EC 3.1.4.17) (Cyclic GMP-stimulated phosphodiesterase) (CGS-PI	0.56
DUSP16	Dual specificity protein phosphatase 16 (EC 3.1.3.48) (EC 3.1.3.16) (Mitogen-activated protein kinase phosphatase 7	0.56
SULT2A1	Bile salt sulfotransferase (EC 2.8.2.14) (Hydroxysteroid Sulfotransferase) (HST) (Dehydroepiandrosterone sulfotran	0.57
SORCS1	VPS10 domain-containing receptor SorCS1 precursor (hSorCS)	0.57
GSTA1	Glutathione S-transferase A1 (EC 2.5.1.18) (GTH1) (HA subunit 1) (GST- epsilon) (GSTA1-1) (GST class-alpha me	0.57
MIR17HG	MIR17 host gene (non-protein coding), MicroRNAs (miRNAs) are small regulatory RNAs that control gene express	0.58
