# Supplementary material for: Knockdown of SF-1 and RNF31 Affects Components of Steroidogenesis, TGFβ, and Wnt/β-catenin Signaling in Adrenocortical Carcinoma Cells
Source: PLoS One. 2012 Mar 9;7(3):e32080. doi: 10.1371/journal.pone.0032080 (PMC3302881; doi:10.1371/journal.pone.0032080)
Supplement: Table S3 — 35 most upregugulated genes in SF-1 RNAi+cAMP-treated cells. (PDF) [file pone.0032080.s003.pdf]

Supplementary table 3. 35 most upregugulated genes in SF-1 RNAi+cAMP-treated cells

Gene Symbol	Description	Fold Change
TFPI2	Tissue factor pathway inhibitor 2 precursor (TFPI-2)	8.06
VGF	Neurosecretory protein VGF precursor.	5.27
EGFR	Epidermal growth factor receptor precursor (EC 2.7.10.1)	5.08
PTPRN	Receptor-type tyrosine-protein phosphatase-like N precursor (R-PTP-N)	4.48
CPLX2	Complexin-2 (Complexin II) (CPX II) (Synaphin-1).	4.47
AMDHD1	amidohydrolase domain containing 1	4.38
MTSS1	Metastasis suppressor protein 1 (Missing in metastasis protein)	4.26
ETV5	ETS translocation variant 5 (Ets-related protein ERM).	4.13
STAR	Steroidogenic acute regulatory protein, mitochondrial precursor	4.02
CYP17A1	Cytochrome P450 17A1 (EC 1.14.99.9) (CYPXVII) (P450-C17)	3.96
NPR1	Atrial natriuretic peptide receptor A precursor (ANP-A) (ANPRA)	3.95
CSN1S1	Alpha-S1-casein precursor [Contains: Casoxin-D]	3.93
NR4A1 / NGFIB	Orphan nuclear receptor NR4A1 (Orphan nuclear receptor HMR)	3.50
GNG11	Guanine nucleotide-binding protein G(I)/G(S)/G(O) gamma-11 subunit precursor.	3.48
SCG2	Secretogranin-2 precursor	3.44
PTP4A1	Protein tyrosine phosphatase type IVA protein 1 (EC 3.1.3.48)	3.40
CPLX2	Complexin-2 (Complexin II) (CPX II) (Synaphin-1)	3.32
OGT	UDP-N-acetylglucosamine--peptide N-acetylglucosaminyltransferase 110 kDa subunit	3.27
GK	Glycerol kinase (EC 2.7.1.30) (ATP:glycerol 3-phosphotransferase) (Glycerokinase) (GK).	3.11
TESK1	Dual specificity testis-specific protein kinase 1 (EC 2.7.12.1) (Testicular protein kinase 1).	3.11
GRAMD1B	GRAMD1B protein	3.10
CREM	cAMP-responsive element modulator.	3.04
CYP21A2	Cytochrome P450 21 (EC 1.14.99.10) (Cytochrome P450 XXI)	3.03
RHOB	Rho-related GTP-binding protein RhoB precursor (H6).	3.02
CYP17A1	Cytochrome P450 17A1 (EC 1.14.99.9) (CYPXVII) (P450-C17) (P450c17)	2.98
NR4A1 / NGFIB	NGFIB	2.93
GJB2	Gap junction beta-2 protein (Connexin-26) (Cx26)	2.91
CYP21A2	Cytochrome P450 21 (EC 1.14.99.10) (Cytochrome P450 XXI)	2.87
SPINK5	Serine protease inhibitor Kazal-type 5 precursor (Lympho-epithelial Kazal-type-related inhibitor)	2.86
RAN	GTP-binding nuclear protein Ran (GTPase Ran) (Ras-like protein TC4)	2.85
PDE7B	cAMP-specific 3',5'-cyclic phosphodiesterase 7B (EC 3.1.4.17).	2.81
NP_060712.2	CDNA FLJ10847 fis, clone NT2RP4001379	2.78
MUM1L1	MUM1-like protein 1 (Mutated melanoma-associated antigen 1-like protein 1).	2.77
PREI3	Preimplantation protein 3 (Mps one binder kinase activator-like 3) (Mob1 homolog 3) (Mob3)	2.76
SFRP1	Secreted frizzled-related protein 1 precursor (sFRP-1) (Frizzled- related protein 1) (FRP-1)	2.76
