# Supplementary material for: Knockdown of SF-1 and RNF31 Affects Components of Steroidogenesis, TGFβ, and Wnt/β-catenin Signaling in Adrenocortical Carcinoma Cells
Source: PLoS One. 2012 Mar 9;7(3):e32080. doi: 10.1371/journal.pone.0032080 (PMC3302881; doi:10.1371/journal.pone.0032080)
Supplement: Table S4 — 35 most downregugulated genes in SF-1 RNAi+cAMP-treated cells. (PDF) [file pone.0032080.s004.pdf]

Supplementary table 4. 35 most downregugulated genes in SF-1 RNAi+cAMP-treated cells

Gene Symbol	Description	Fold Change
GABBR2	Gamma-aminobutyric acid type B receptor, subunit 2 precursor (GABA-B receptor 2)	0.29
PGM2L1	Phosphoglucomutase-2-like 1 (EC 5.4.2.2) (PMMLP). [Source:Uniprot/SWISSPROT;Acc:Q6PCE3]	0.31
DBP	D site-binding protein (Albumin D box-binding protein	0.32
CTTNBP2	Cortactin-binding protein 2 (CortBP2).	0.33
PDGFD	Platelet-derived growth factor D precursor (PDGF D) (Iris-expressed growth factor)	0.33
GPR37	Probable G-protein coupled receptor 37 precursor (Endothelin B receptor-like protein 1)	0.33
Q9H599_HUMAN	OTTHUMP00000030295 (Fragment	0.34
FAM5B	Protein FAM5B precursor (BMP/retinoic acid-inducible neural-specific protein 2)	0.37
Q9NU38_HUMAN	BA395L14.5 (Novel phosphoglucomutase like protein) (Fragment).	0.37
PCDH18	Protocadherin-18 precursor.	0.39
PPAP2A	Lipid phosphate phosphohydrolase 1 (EC 3.1.3.4) (Phosphatidic acid phosphatase 2a)	0.39
SLC2A13	Proton myo-inositol cotransporter (H(+)-myo-inositol cotransporter) (Hmit)]	0.39
GRIN3A	Glutamate [NMDA] receptor subunit 3A precursor (N-methyl-D-aspartate receptor subtype NR3A)	0.39
TXNIP	Thioredoxin-interacting protein (Vitamin D3 up-regulated protein 1) (Thioredoxin-binding protein 2).	0.39
CD36	Platelet glycoprotein 4 (Platelet glycoprotein IV) (GPIV) (Glycoprotein IIIb)	0.40
NUDT10	Diphosphoinositol polyphosphate phosphohydrolase 3 alpha (EC 3.6.1.52)	0.40
SLC40A1	Solute carrier family 40 member 1 (Ferroportin-1) (Iron-regulated transporter 1)	0.40
CAV2	Caveolin-2	0.41
PGM5	Phosphoglucomutase-like protein 5 (Phosphoglucomutase-related protein) (PGM-RP) (Aciculin).	0.42
CTNNA2	Catenin alpha-2 (Alpha-catenin-related protein) (Alpha N-catenin)	0.42
GSTA2	Glutathione S-transferase A2 (EC 2.5.1.18) (GTH2) (HA subunit 2) (GST- gamma) (GSTA2-2)	0.42
ALDH1A1	Retinal dehydrogenase 1 (EC 1.2.1.36) (RalDH1) (RALDH 1)	0.43
KCNK2	Potassium channel subfamily K member 2 (Outward rectifying potassium channel protein TREK-1)	0.43
ST3GAL6	Type 2 lactosamine alpha-2,3-sialyltransferase (EC 2.4.99.-)	0.44
OSBPL6	Oxysterol-binding protein-related protein 6 (OSBP-related protein 6) (ORP-6).	0.44
C4orf18	AD021 protein (C4orf18 protein) (Hypothetical protein DKFZp434L142)	0.44
TBA3_HUMAN	Tubulin alpha-3 chain (Alpha-tubulin 3) (Tubulin B-alpha-1)	0.44
FGF13	Fibroblast growth factor 13 (FGF-13) (Fibroblast growth factor homologous factor 2) (FHF-2)	0.44
IQGAP2	Ras GTPase-activating-like protein IQGAP2.	0.45
SMAD9	Mothers against decapentaplegic homolog 9 (SMAD 9) (Mothers against DPP homolog 9) (Smad9)	0.45
NR1D2	Orphan nuclear receptor NR1D2 (Rev-erb-beta) (EAR-1R) (Orphan nuclear hormone receptor BD73)	0.45
SLC44A1	Choline transporter-like protein 1 (Solute carrier family 44 member 1) (CD92 antigen) (CDw92).	0.45
CTNNA2	Catenin alpha-2 (Alpha-catenin-related protein) (Alpha N-catenin).	0.46
FIBCD1	fibrinogen C domain containing 1	0.46
ACPP	Prostatic acid phosphatase precursor (EC 3.1.3.2)	0.46
