# Supplementary material for: Knockdown of SF-1 and RNF31 Affects Components of Steroidogenesis, TGFβ, and Wnt/β-catenin Signaling in Adrenocortical Carcinoma Cells
Source: PLoS One. 2012 Mar 9;7(3):e32080. doi: 10.1371/journal.pone.0032080 (PMC3302881; doi:10.1371/journal.pone.0032080)
Supplement: Table S5 — 35 most upregulated genes in cAMP-treated cells. (PDF) [file pone.0032080.s005.pdf]

Supplementary table 5. 35 most upregulated genes in cAMP-treated cells

Gene Symbol	Description	Fold Change
TFPI2	Tissue factor pathway inhibitor 2 precursor (TFPI-2) (Placental protein 5) (PP5)	8.18
VGF	Neurosecretory protein VGF precursor	5.64
NOR1, MINOR	NOR1, MINOR	5.04
STAR	Steroidogenic acute regulatory protein, mitochondrial precursor (StAR) (StARD1)	4.94
PRKAR2B	cAMP-dependent protein kinase type II-beta regulatory subunit	4.55
CSN1S1	Alpha-S1-casein precursor [Contains: Casoxin-D]	4.37
TESK1	Dual specificity testis-specific protein kinase 1 (EC 2.7.12.1) (Testicular protein kinase 1)	4.30
NR4A3 / NOR1	Orphan nuclear receptor NR4A3 (Nuclear hormone receptor NOR-1)	4.15
MTSS1	Metastasis suppressor protein 1 (Missing in metastasis protein) (Metastasis suppressor YGL-1)	4.13
ALDH3A2	Fatty aldehyde dehydrogenase (EC 1.2.1.3) (Aldehyde dehydrogenase, microsomal)	3.99
PAPSS2	Bifunctional 3'-phosphoadenosine 5'-phosphosulfate synthetase 2 (PAPS synthetase 2)	3.89
CAMK2N1	calcium/calmodulin-dependent protein kinase II inhibitor 1	3.89
ETV5	ETS translocation variant 5 (Ets-related protein ERM)	3.74
CYP17A1	cytochrome P450, family 17, subfamily A, polypeptide 1	3.73
RAN	GTP-binding nuclear protein Ran (GTPase Ran) (Ras-like protein TC4)	3.48
IKBKAP	IkappaB kinase complex-associated protein (IKK complex-associated protein) (p150)	3.47
CYP21A2	Cytochrome P450 21 (EC 1.14.99.10) (Cytochrome P450 XXI) (Steroid 21- hydroxylase)	3.46
PTPN3	Tyrosine-protein phosphatase non-receptor type 3 (EC 3.1.3.48)	3.36
NXN	Nucleoredoxin	3.29
KIF3A	Kinesin-like protein KIF3A (Microtubule plus end-directed kinesin motor 3A)	3.26
TAL1	T-cell acute lymphocytic leukemia-1 protein (TAL-1 protein) (Stem cell protein)	3.25
NR4A1 / NGFIB	Orphan nuclear receptor NR4A1 (Orphan nuclear receptor HMR)	3.15
ALPL	Alkaline phosphatase, tissue-nonspecific isozyme precursor (EC 3.1.3.1)	3.14
GNG11	Guanine nucleotide-binding protein G(I)/G(S)/G(O) gamma-11 subunit precursor	3.10
GK	Glycerol kinase (EC 2.7.1.30) (ATP:glycerol 3-phosphotransferase) (Glycerokinase) (GK)	3.02
PTPRN	Receptor-type tyrosine-protein phosphatase-like N precursor (R-PTP-N)	3.04
SYMPK	Symplekin	3.02
NEK6	Serine/threonine-protein kinase Nek6 (EC 2.7.11.1) (NimA-related protein kinase 6)	3.00
RRS1	Ribosome biogenesis regulatory protein homolog	2.94
CREM	cAMP-responsive element modulator	2.92
STX2	Syntaxin-2 (Epimorphin)	2.83
METTL7B	Methyltransferase-like protein 7B precursor (EC 2.1.1.-)	2.79
NP_060712.2	CDNA FLJ10847 fis, clone NT2RP4001379.	2.77
MOBKL3	Mps one binder kinase activator-like 3 (Mob1 homolog 3) (Mob3) (Class II mMOB1) (2C4D)	2.76
RHOB	Rho-related GTP-binding protein RhoB precursor (H6). [Source:Uniprot/SWISSPROT;Acc:P62745]	2.76
