# Supplementary material for: Knockdown of SF-1 and RNF31 Affects Components of Steroidogenesis, TGFβ, and Wnt/β-catenin Signaling in Adrenocortical Carcinoma Cells
Source: PLoS One. 2012 Mar 9;7(3):e32080. doi: 10.1371/journal.pone.0032080 (PMC3302881; doi:10.1371/journal.pone.0032080)
Supplement: Table S6 — 35 most downregulated genes in cAMP-treated cells. (PDF) [file pone.0032080.s006.pdf]

Supplementary table 6. 35 most downregulated genes in cAMP-treated cells

Gene Symbol	Description	Fold Change
ZNF385	Zinc finger protein 385 (Hematopoietic zinc finger protein) (Retinal zinc finger protein).	0.12
JARID1A	Jumonji/ARID domain-containing protein 1A (Retinoblastoma-binding protein 2) (RBBP-2).	0.19
FKBP9L	FK506 binding protein 9-like	0.19
NRGN	Neurogranin (Ng) (RC3)	0.20
ETFB	Electron transfer flavoprotein subunit beta (Beta-ETF)	0.20
INTS12	Integrator complex subunit 12 (Int12) (PHD finger protein 22)	0.21
PABPC5	Polyadenylate-binding protein 5 (Poly(A)-binding protein 5) (PABP 5	0.23
MLEC	Malectin (KIAA0152)	0.23
SCRG1_HUMAN	Scrapie-responsive protein 1 precursor (ScRG-1)	0.25
ELOVL1	Elongation of very long chain fatty acids protein 1	0.26
CTAGE5	Cutaneous T-cell lymphoma-associated antigen 5 (cTAGE-5 protein) (cTAGE family member 5)	0.27
HCFC1R1	host cell factor C1 regulator 1 (XPO1 dependant) isoform 2	0.27
SERINC5	developmentally regulated protein TPO1	0.28
DBP	D site-binding protein (Albumin D box-binding protein) (Albumin D- element-binding protein) (TAXREB302)	0.30
ABCB6	Mitochondrial ATP-binding cassette sub-family B member 6 (Mitochondrial ABC transporter 3)	0.31
CLDN1	Claudin-1 (Senescence-associated epithelial membrane protein)	0.31
REEP6	Receptor expression-enhancing protein 6 (Polyposis locus protein 1- like 1)	0.32
NRCAM	Neuronal cell adhesion molecule precursor (Nr-CAM) (NgCAM-related cell adhesion molecule)	0.35
PRSS23	Serine protease 23 precursor (EC 3.4.21.-) (Putative secreted protein ZSIG13)	0.36
FGF13	Fibroblast growth factor 13 (FGF-13) (Fibroblast growth factor homologous factor 2) (FHF-2).	0.36
ZNF403	Protein ZNF403 (Laryngeal carcinoma-related protein 1)	0.37
CHN1	N-chimaerin (NC) (N-chimerin) (Alpha chimerin) (A-chimaerin) (Rho GTPase-activating protein 2)	0.37
APCDD1	Protein APCDD1 precursor (Adenomatosis polyposis coli down-regulated 1 protein)	0.37
IQGAP2	Ras GTPase-activating-like protein IQGAP2	0.38
GSTA1	Glutathione S-transferase A1 (EC 2.5.1.18) (GTH1) (HA subunit 1) (GST- epsilon) (GSTA1-1)	0.39
ACPL2	acid phosphatase-like 2	0.39
CEP78	CDNA FLJ12643 fis, clone NT2RM4001969, moderately similar to R.norvegicus IP63 protein. (Fragment)	0.39
APLN	apelin, AGTRL1 ligand (APLN)	0.39
SV2B	Synaptic vesicle glycoprotein 2B	0.40
THRA	Thyroid hormone receptor alpha (C-erbA-alpha) (c-erbA-1) (EAR-7)	0.40
GSTA2	Glutathione S-transferase A2 (EC 2.5.1.18) (GTH2) (HA subunit 2) (GST- gamma) (GSTA2-2)	0.40
LRRC61	Leucine-rich repeat-containing protein 61	0.41
LOC729468	similar to Putative PGM5-like protein 1	0.41
GABBR2	Gamma-aminobutyric acid type B receptor, subunit 2 precursor (GABA-B receptor 2) (GABA-B-R2)	0.42
CCDC141	coiled-coil domain containing 141	0.43
