# Supplementary material for: Knockdown of SF-1 and RNF31 Affects Components of Steroidogenesis, TGFβ, and Wnt/β-catenin Signaling in Adrenocortical Carcinoma Cells
Source: PLoS One. 2012 Mar 9;7(3):e32080. doi: 10.1371/journal.pone.0032080 (PMC3302881; doi:10.1371/journal.pone.0032080)
Supplement: Table S7 — 35 most upregulated genes in RNF31 RNAi-treated cells. (PDF) [file pone.0032080.s007.pdf]

Supplementary table 7. 35 most upregulated genes in RNF31 RNAi-treated cells

Gene Symbol	Description	Fold Change
CYP19A1	cytochrome P450, family 19, subfamily A, polypeptide 1 Aromatase	7.47
NPY1R	neuropeptide Y receptor Y1	3.19
SPINK5	serine peptidase inhibitor, Kazal type 5	2.88
NXN	nucleoredoxin	2.84
ETV5	ets variant 5	2.80
LOC390811	polymerase (RNA) III (DNA directed) polypeptide K, 12.3 kDa pseudogene	2.73
CA2	carbonic anhydrase II	2.69
CCL22	chemokine (C-C motif) ligand 22	2.65
CXCR4	chemokine (C-X-C motif) receptor 4	2.58
LOC728510	hypothetical LOC728510	2.53
CYP21A2	cytochrome P450, family 21, subfamily A, polypeptide 2	2.51
GNAI1	guanine nucleotide binding protein (G protein), alpha inhibiting activity polypeptide 1	2.29
ENC1	ectodermal-neural cortex 1 (with BTB-like domain)	2.25
HSD3B1	hydroxy-delta-5-steroid dehydrogenase, 3 beta- and steroid delta-isomerase 1	2.08
KCNN2	potassium intermediate/small conductance calcium-activated channel, subfamily N, member 2	2.07
PLK2	polo-like kinase 2	2.06
TRIB2	tribbles homolog 2	2.05
AMDHD1	amidohydrolase domain containing 1	2.05
UGCG	UDP-glucose ceramide glucosyltransferase	1.99
TUBB4	tubulin, beta 4	1.98
NOV	nephroblastoma overexpressed gene	1.95
ALDH3A2	aldehyde dehydrogenase 3 family, member A2	1.92
NEK6	NIMA (never in mitosis gene a)-related kinase 6	1.91
ITGA1	integrin, alpha 1	1.89
MTSS1	metastasis suppressor 1	1.89
VEGFC	vascular endothelial growth factor C	1.89
PLD5	phospholipase D family, member 5	1.88
C6orf176	chromosome 6 open reading frame 176	1.86
STAR	steroidogenic acute regulatory protein	1.86
MC2R	melanocortin 2 receptor	1.85
C9orf84	chromosome 9 open reading frame 84	1.84
PDK1	pyruvate dehydrogenase kinase, isozyme 1	1.82
DUSP4	dual specificity phosphatase 4	1.81
PAPSS2	3'-phosphoadenosine 5'-phosphosulfate synthase 2	1.79
STON2	stonin 2	1.78
