# Supplementary material for: Knockdown of SF-1 and RNF31 Affects Components of Steroidogenesis, TGFβ, and Wnt/β-catenin Signaling in Adrenocortical Carcinoma Cells
Source: PLoS One. 2012 Mar 9;7(3):e32080. doi: 10.1371/journal.pone.0032080 (PMC3302881; doi:10.1371/journal.pone.0032080)
Supplement: Table S8 — 35 most downregulated genes in RNF31 RNAi-treated cells. (PDF) [file pone.0032080.s008.pdf]

Supplementary table 8. 35 most downregulated genes in RNF31 RNAi-treated cells

Gene Symbol	Description	Fold change
ANKFN1	ankyrin-repeat and fibronectin type III domain containing 1	0.40
UST	uronyl-2-sulfotransferase	0.51
ITGA8	integrin, alpha 8	0.53
TMED5	transmembrane emp24 protein transport domain containing 5	0.53
DMD	dystrophin	0.53
RCN2	reticulocalbin 2, EF-hand calcium binding domain	0.54
STC1	stanniocalcin 1	0.54
ISM1	isthmin 1 homolog	0.54
FBN2	fibrillin 2	0.54
FGF13	fibroblast growth factor 13	0.55
COL18A1	collagen, type XVIII, alpha 1	0.55
CTAGE5	CTAGE family, member 5	0.55
GPR64	G protein-coupled receptor 64	0.55
YWHAZ	tyrosine 3-monooxygenase/tryptophan 5-monooxygenase activation protein, zeta polypeptide	0.56
APCDD1	adenomatosis polyposis coli down-regulated 1	0.56
ACPL2	acid phosphatase-like 2	0.57
FST	follistatin	0.57
KCTD12	potassium channel tetramerisation domain containing 12	0.57
NEK7	NIMA (never in mitosis gene a)-related kinase 7	0.59
KLK4	kallikrein-related peptidase 4	0.60
ANKRD27	ankyrin repeat domain 27 (VPS9 domain)	0.60
UBE2D1	ubiquitin-conjugating enzyme E2D 1 (UBC4/5 homolog, yeast)	0.60
FNDC3B	fibronectin type III domain containing 3B	0.60
AP3S1	adaptor-related protein complex 3, sigma 1 subunit	0.60
DACH2	dachshund homolog 2	0.60
NEFL	neurofilament, light polypeptide	0.60
NRCAM	neuronal cell adhesion molecule	0.61
MITF	microphthalmia-associated transcription factor	0.61
SYTL2	synaptotagmin-like 2	0.61
**RNF31**	**ring finger protein 31**	0.61
MME	membrane metallo endopeptidase	0.61
WDR19	WD repeat domain 19	0.61
EMP2	epithelial membrane protein 2	0.61
PDZRN3	PDZ domain containing ring finger 3	0.62
SLC44A5	solute carrier family 44, member 5	0.62
