# Supplementary material for: Knockdown of SF-1 and RNF31 Affects Components of Steroidogenesis, TGFβ, and Wnt/β-catenin Signaling in Adrenocortical Carcinoma Cells
Source: PLoS One. 2012 Mar 9;7(3):e32080. doi: 10.1371/journal.pone.0032080 (PMC3302881; doi:10.1371/journal.pone.0032080)
Supplement: Table S9 — 35 most upregugulated genes inRNF31 RNAi+cAMP treated cells. (PDF) [file pone.0032080.s009.pdf]

Supplementary table 9. 35 most upregugulated genes inRNF31 RNAi+cAMP treated cells

Gene Symbol	Description	Fold Change
UTS2	urotensin 2	18.73
EGFR	Epidermal growth factor receptor	8.26
IL7R	Interleukin 7 receptor	5.81
AMDHD1	amidohydrolase domain containing 1	5.05
TFPI2	tissue factor pathway inhibitor 2	4.93
SFRP1	secreted frizzled-related protein 1	4.58
CYP17A1	cytochrome P450, family 17, subfamily a, polypeptide 1	4.10
MAN1A1	mannosidase, alpha, class 1A, member 1	3.85
NPR1	natriuretic peptide receptor A/guanylate cyclase A (atrionatriuretic peptide receptor A)	3.71
CHRNA7	cholinergic receptor, nicotinic, alpha polypeptide 7	3.51
MTSS1	metastasis suppressor 1	3.46
SLC25A19	mitochondrial thiamine pyrophosphate carrier	3.44
CAMK2N1	calcium/calmodulin-dependent protein kinase II inhibitor 1	3.42
LONRF2	LON peptidase N-terminal domain and ring finger 2	3.31
VGF	VGF nerve growth factor inducible	3.23
CSN1S1	casein alpha s1	3.22
FRMD5	FERM domain containing 5	3.16
CGA	glycoprotein hormones, alpha polypeptide	3.10
PRKAR2B	protein kinase, cAMP-dependent, regulatory, type II, beta	3.01
KCND2	potassium voltage-gated channel, Shal-related subfamily, member 2	3.01
STX2	syntaxin 2	2.99
NR4A1	(NGFIB, Nur77)	2.85
FAM46A	family with sequence similarity 46, member A	2.83
NR4A3	MINOR, NOR1	2.82
GJB2	gap junction protein, beta 2	2.81
ESR2	estrogen receptor 2 (ER beta)	2.81
PTPRN	protein tyrosine phosphatase, receptor type, N	2.80
C6orf176	chromosome 6 open reading frame 176	2.79
IKBKAP	inhibitor of kappa light polypeptide gene enhancer in B-cells, kinase complex-associated protein	2.74
SERPINE2	serpin peptidase inhibitor, clade E	2.74
STAR	steroidogenic acute regulatory protein	2.70
PAPSS2	3'-phosphoadenosine 5'-phosphosulfate synthase 2	2.69
ALDH3A2	aldehyde dehydrogenase 3 family, member A2	2.67
SPRY2	sprouty homolog 2	2.66
