# Supplementary material for: Knockdown of SF-1 and RNF31 Affects Components of Steroidogenesis, TGFβ, and Wnt/β-catenin Signaling in Adrenocortical Carcinoma Cells
Source: PLoS One. 2012 Mar 9;7(3):e32080. doi: 10.1371/journal.pone.0032080 (PMC3302881; doi:10.1371/journal.pone.0032080)
Supplement: Table S10 — 35 most downregugulated genes inRNF31 RNAi+cAMP treated cells. (PDF) [file pone.0032080.s010.pdf]

Supplementary table 10. 35 most downregugulated genes inRNF31 RNAi+cAMP treated cells

Gene Symbol	Description	Fold Change
ISM1	isthmin-1	0.20
ANKFN1	ankyrin-repeat and fibronectin type III domain containing 1	0.27
NRCAM	neuronal cell adhesion molecule	0.30
FAM5B	family with sequence similarity 5, member B	0.30
IQGAP2	IQ motif containing GTPase activating protein 2	0.32
CLDN1	claudin 1	0.33
NOV	nephroblastoma overexpressed gene	0.33
KLHL5	kelch-like 5	0.34
TXNIP	thioredoxin interacting protein	0.34
CTAGE5	CTAGE family, member 5	0.35
SULT2A1	sulfotransferase family 2A, (DHEA)-preferring, member 1	0.35
HIST1H2AK	histone cluster 1, H2ak	0.35
GABBR2	gamma-aminobutyric acid (GABA) B receptor, 2	0.36
PLAGL1	pleiomorphic adenoma gene-like 1	0.37
FIBCD1	fibrinogen C domain containing 1	0.37
DBP	D site of albumin promoter (albumin D-box) binding protein	0.37
LOC729468	putative PGM5-like protein 1-like	0.38
CHN1	chimerin (chimaerin) 1	0.39
FAM198B	family with sequence similarity 198, member B	0.40
PDE2A	phosphodiesterase 2A, cGMP-stimulated	0.42
PPAP2A	phosphatidic acid phosphatase type 2A	0.42
ACPL2	acid phosphatase-like 2	0.42
SLC35D1	UDPglucuronic acid/UDP-N-acetylgalactosamine dual transporter	0.42
GRIN3A	glutamate receptor, ionotropic, N-methyl-D-aspartate 3A	0.42
HIST2H2BF,HIST2H2BA	histone cluster 2, H2b	0.42
C5orf13	chromosome 5 open reading frame 13	0.43
PRSS23	protease, serine, 23	0.43
TRPM3	transient receptor potential cation channel	0.43
FAM198B	family with sequence similarity 198, member B	0.43
SERINC5	serine incorporator 5	0.43
ST3GAL6	ST3 beta-galactoside alpha-2,3-sialyltransferase 6	0.43
HSPB7	heat shock 27kDa protein family, member 7	0.43
GSTA2	glutathione S-transferase A2	0.43
CAV2	caveolin 2	0.44
FGF13	Fibroblast growth factor 13	0,44
