# Supplementary material for: Knockdown of SF-1 and RNF31 Affects Components of Steroidogenesis, TGFβ, and Wnt/β-catenin Signaling in Adrenocortical Carcinoma Cells
Source: PLoS One. 2012 Mar 9;7(3):e32080. doi: 10.1371/journal.pone.0032080 (PMC3302881; doi:10.1371/journal.pone.0032080)
Supplement: Table S11 — 50 first hits in DAVID functional annotation chart of siSF-1 microarray. (PDF) [file pone.0032080.s011.pdf]

Supplementary table 11. 50 first hits in DAVID functional annotation chart of siSF-1 microarray

	Category	Term	Count	P-Value
1	SP_PIR_KEYWORDS	phosphoprotein	157	3.82E-06
2	GOTERM_CC_FAT	GO:0044459~plasma membrane part	64	5.37E-05
3	GOTERM_BP_FAT	GO:0051094~positive regulation of developmental process	16	9.15E-05
4	GOTERM_CC_FAT	GO:0005626~insoluble fraction	31	2.05E-04
5	GOTERM_BP_FAT	GO:0010557~positive regulation of macromolecule biosynthetic process	25	3.69E-04
6	GOTERM_CC_FAT	GO:0005912~adherens junction	11	4.61E-04
7	GOTERM_BP_FAT	GO:0016055~Wnt receptor signaling pathway	10	4.74E-04
8	GOTERM_BP_FAT	GO:0045597~positive regulation of cell differentiation	13	5.98E-04
9	GOTERM_BP_FAT	GO:0009725~response to hormone stimulus	17	6.01E-04
10	SP_PIR_KEYWORDS	steroidogenesis	4	6.21E-04
11	GOTERM_BP_FAT	GO:0010033~response to organic substance	26	6.38E-04
12	GOTERM_BP_FAT	GO:0001649~osteoblast differentiation	6	7.20E-04
13	GOTERM_MF_FAT	GO:0004114~3',5'-cyclic-nucleotide phosphodiesterase activity	5	7.35E-04
14	GOTERM_BP_FAT	GO:0010604~positive regulation of macromolecule metabolic process	29	7.65E-04
15	GOTERM_CC_FAT	GO:0000267~cell fraction	35	8.30E-04
16	GOTERM_MF_FAT	GO:0004112~cyclic-nucleotide phosphodiesterase activity	5	8.63E-04
17	GOTERM_BP_FAT	GO:0009891~positive regulation of biosynthetic process	25	8.69E-04
18	SP_PIR_KEYWORDS	membrane	128	0.001011
19	GOTERM_CC_FAT	GO:0070161~anchoring junction	11	0.001033
20	GOTERM_BP_FAT	GO:0007548~sex differentiation	10	0.001178
21	GOTERM_CC_FAT	GO:0005886~plasma membrane	90	0.001240
22	GOTERM_BP_FAT	GO:0010324~membrane invagination	12	0.001466
23	GOTERM_BP_FAT	GO:0006897~endocytosis	12	0.001466
24	GOTERM_BP_FAT	GO:0007267~cell-cell signaling	22	0.001557
25	GOTERM_BP_FAT	GO:0031328~positive regulation of cellular biosynthetic process	24	0.001610
26	GOTERM_BP_FAT	GO:0009719~response to endogenous stimulus	17	0.001691
27	SP_PIR_KEYWORDS	lipoprotein	22	0.001784
28	GOTERM_BP_FAT	GO:0042127~regulation of cell proliferation	26	0.002164
29	GOTERM_BP_FAT	GO:0043627~response to estrogen stimulus	8	0.002185
30	GOTERM_CC_FAT	GO:0030424~axon	10	0.002197
31	GOTERM_BP_FAT	GO:0048732~gland development	9	0.002272
32	GOTERM_CC_FAT	GO:0005925~focal adhesion	8	0.002305
33	GOTERM_MF_FAT	GO:0008047~enzyme activator activity	15	0.002316
34	GOTERM_BP_FAT	GO:0045935~pos. reg. of nucleob., nucleos., nucleot. and nucleic acid meta	22	0.002492
35	GOTERM_CC_FAT	GO:0005624~membrane fraction	27	0.002560
36	GOTERM_BP_FAT	GO:0007584~response to nutrient	9	0.002848
37	GOTERM_CC_FAT	GO:0005924~cell-substrate adherens junction	8	0.002866
38	SP_PIR_KEYWORDS	Rotamase	5	0.002874
39	GOTERM_MF_FAT	GO:0008081~phosphoric diester hydrolase activity	7	0.003496
40	GOTERM_BP_FAT	GO:0051173~positive regulation of nitrogen compound metabolic process	22	0.003623
41	GOTERM_BP_FAT	GO:0001503~ossification	8	0.003644
42	SP_PIR_KEYWORDS	wnt signaling pathway	8	0.003649
43	GOTERM_MF_FAT	GO:0003755~peptidyl-prolyl cis-trans isomerase activity	5	0.003823
44	GOTERM_CC_FAT	GO:0030055~cell-substrate junction	8	0.003895
45	GOTERM_BP_FAT	GO:0045941~positive regulation of transcription	20	0.003930
46	KEGG_PATHWAY	hsa04350:TGF-beta signaling pathway	7	0.004162
47	GOTERM_BP_FAT	GO:0009214~cyclic nucleotide catabolic process	3	0.004198
48	GOTERM_BP_FAT	GO:0042989~sequestering of actin monomers	3	0.004198
49	GOTERM_BP_FAT	GO:0043277~apoptotic cell clearance	3	0.004198
50	GOTERM_MF_FAT	GO:0016859~cis-trans isomerase activity	5	0.004634
